# Reversible massive ascites from amiodarone hepatotoxicity

**DOI:** 10.1093/omcr/omag007

**Published:** 2026-02-24

**Authors:** Yohei Nishimura, Yu Horiuchi, Kengo Tanabe

**Affiliations:** Division of Cardiology, Mitsui Memorial Hospital, Kanda-Izumicho 1, Chiyoda-ku, Tokyo 101-8643, Japan; Division of Cardiology, Mitsui Memorial Hospital, Kanda-Izumicho 1, Chiyoda-ku, Tokyo 101-8643, Japan; Division of Cardiology, Mitsui Memorial Hospital, Kanda-Izumicho 1, Chiyoda-ku, Tokyo 101-8643, Japan

**Keywords:** amiodarone, hepatotoxicity, ascites

**Figure 1:**
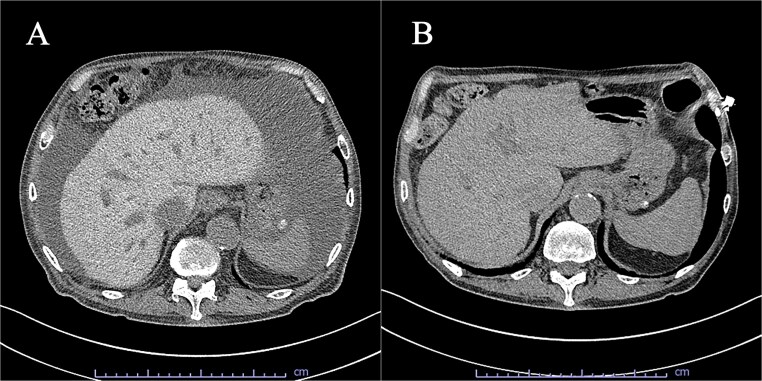
Abdominal computed tomography showing (A) increased hepatic attenuation and ascites and (B) resolution of ascites after discontinuation of amiodarone.

A 76-year-old man presented with progressive distension of the abdomen. He had a history of heart failure with reduced ejection fraction due to dilated cardiomyopathy, severe tricuspid regurgitation, and implantation of a cardiac resynchronization therapy defibrillator. He had been receiving guideline-directed medical therapy for heart failure and oral amiodarone 200 mg daily for approximately one year for sustained ventricular tachycardia. Physical examination revealed ascites, confirmed by a positive fluid wave test, and bilateral pitting edema in the lower extremities. Ophthalmologic examination revealed corneal deposits consistent with amiodarone-induced keratopathy. Laboratory tests showed mildly elevated hepatic enzyme levels, normal thyroid function, and serum levels of amiodarone and its metabolite within the therapeutic range. No signs of amiodarone-induced pulmonary toxicity were observed. Clinical findings were suggestive of right-sided heart failure. Intensified diuretic therapy was administered, but the massive ascites persisted. Abdominal computed tomography (CT) revealed a diffusely hyperattenuating liver (102 Hounsfield units [HU]) accompanied by massive ascites (Fig. 1A). Liver attenuation on CT prior to amiodarone initiation a year earlier was within the normal range (60 HU). Based on these findings, we suspected amiodarone hepatotoxicity. Following detailed risk–benefit discussion with the patient, amiodarone was discontinued. Subsequently, the ascites resolved completely and liver enzyme levels improved (Fig. 1B).

Amiodarone hepatotoxicity is a rare condition. Asymptomatic elevations in liver enzymes are observed in 15%–30% of patients receiving amiodarone, whereas clinically significant hepatotoxicity occurs in less than 3% of patients [1, 2]. Amiodarone and its metabolites are highly lipophilic and accumulate in the hepatic tissue, resulting in increased CT attenuation values that indicate hepatic deposition [3]. Ascites is an uncommon manifestation of amiodarone hepatotoxicity and may resolve with drug cessation. This diagnosis should be considered in patients receiving long-term amiodarone therapy who present with unexplained ascites.
